# Book Notices and Reviews

**Published:** 1864-01

**Authors:** 


### BOOK NOTICES AND REVIEWS.

*Outlines of the Chief Camp Diseases of the United States Armies as observed during the Present War—A Practical Contribution to Military Medicine.* By JOSEPH JANVIER WOODWARD, M. D., Ass't. Surgeon U. S. A.; Member of the Academy of Nat. Sci., Phila.; of the Pathol. Soc., of Phila., etc., etc. Philadelphia: J. B. Lippincott & Co., 1863; pp. 364. $2.50.

The author of the present treatise is already known to the profession by his successful little work—the "Hospital Steward's Manual," and by his connection with the laborious duty of preparing for the Medical Department of the Army "The Medical History of the Rebellion." His official relations have necessarily involved earnest thought upon the subject of Camp Diseases, and with them he had previously acquired practical familiarity by actual service in the field. The book before us is essentially a practical one, and is worthy a place among the field books of the army surgeon, whilst in its pages the civil practitioner will find much valuable information for every day use.

Something of the kind had grown to be a necessity, for the formulæ of the text books on practice are confessedly wholly inadequate to the exigencies of the present time, either in camp or civil life. It is an *avant courier* which heralds the coming of the elaborated results of the grand experiences of the war.

In its general tenor, the book runs parallel with the ideas of Hygienic and Conservative Medicine now generally in vogue—ideas which are a healthy reaction from the formularized specifics, perturbations, and destructive methods of preceding practice. The consolidated facts, afforded by the war, have mercilessly overthrown a host of idolized old dogmas. After alluding to the fears generally entertained at the outset of the war, that our camps would be scourged by the same pestilences which have, again and again, devastated European armies, and that in the change of climate and habits, incident to the progress of the Northern arms Southward, tropical diseases would produce greater havoc than the attacks of the enemy, he gives a brief account of the system of classification of diseases adopted by the Medical Department and some general statements as to the prevalency of each classified form as gathered from the official statistics.

The great conditions determining the character of Camp Diseases are threefold: Malarious, (Roino-Miasmata); Typhoid, (Idio-Miasmata); and Scorbutic. The productive agency of each of these and their blended agency are sketched perspicuously and forcibly. The blended product is known officially as Typho-Malarial Fever, and its history, including treatment, is given in concise and clear terms. Then follow sections upon Intermittent, Simple and Congestive; Chronic Malarial Poisoning; Jaundice; Camp Diarrhœa; Simple Diarrhœa; Acute Entiritis; Acute Dysentery; Chronic Diarrhœa; Camp Measles; Catarrh; Pneumonia; Pseudo-Rheumatic Affections, and, finally, an Appendix.

We strongly recommend the perusal of these chapters to the student of medicine, the army and civil practitioner, as containing a fund of fact, common sense and practical information nowhere else to be found in print. The general adoption of the hygienic and therapeutic methods recommended would go far to lessen both the sickness and mortality of the army, already inconsiderable when compared with any other of even approximate magnitude which has stood upon the face of the planet.

We are gratified to see that the author takes high and broad grounds against the absurd wholesale "Quinine prophylaxis" whilome, so strongly urged by sundry parties. He abjures it almost *totidem verbis* with this Journal when the war began. A.

*A Manual on Extracting Teeth*—Founded on the Anatomy of the parts involved in the operation; the kinds and proper construction of the instruments to be used; the accidents liable to occur from the operation, and the proper remedies to retrieve such accidents. By Abraham Robertson, D.D.S., M. D., Author of Prize Essay on Extracting Teeth, &c. Philadelphia: Lindsay & Blakiston, 1863.

The title page of this little volume explains very fully its objects. From a rapid perusal we have no hesitation in saying the objects have been attained. The importance of the subject will be fully appreciated by every one who is compelled to submit to the unpleasant and painful operation for the removal of a tooth, and the importance also of its extraction with the least exertion, with the least amount of force by the operator, with the least possible injury to the surrounding parts, and consequently with the least amount of present pain and subsequent suffering to the patient.

After the introductory chapter—The Anatomy of the Jaws and Teeth. Pathology of Toothache. Instruments used for extracting teeth, and the proper method of using them. Of lancing the gums. Accidents attendant upon the extraction of teeth, and their remedies. And Anasthetics, each form the subject of a distinct chapter, which may be consulted with advantage by the physician as well as the dentist.

For sale by S. C. Griggs & Co. Price $1,00.

---

*Transactions of the Eighteenth Annual Meeting of the Ohio State Medical Society*—Held at Ohio White Sulphur Springs, June 16, 17, 18, 1863. From E. B. Stevens, M. D., Secretary.

The proceedings of the three day's session of this Society evince a commendable zeal in what its members conceive would advance the science of medicine and elevate the profession. A number of Reports and Essays, of real merit, were read, among which are the following:

The Valedictory Address of the President, J. W. Russell, M. D.

On the Employment of Electricity in Midwifery, by D. S. Gaus, M. D.

On New Remedies, by E. B. Stevens, M. D.

On Mollites Ossium, by N. Dalton, M. D.

On Adipose Tumors, by A. McBride, M. D.

Some of these productions deserve, and did our limits permit should receive a more extended notice—we may recur to them in a future number.

A list of the members is appended, making together a volume of which our neighbors of Ohio may well be proud.

---

*Mental Hygiene.* By J. RAY, M. D. Boston: Ticknor & Fields, 1863.

This work does not claim to be a systematic treatise on *Mental Hygiene*, but to expose the mischievous effects of many practices and customs prevalent in modern society; and to present practical suggestions relative to the attainment of mental soundness and vigor. It contains many facts, and enunciates important principles and suggestions, which will be red with interest and profit by the public and the legislator as well as the physician.

Price $1.00. For sale by S. C. Griggs & Co., 39 & 41 Lake street.

---

*Journals and Pamphlets Received.*—London Lancet, Cincinnati Lancet and Observer, Medical News, Journal of Ophthalmology, Pacific Medical and Surgical Journal, Canada Lancet, Druggists' Circular, Boston Medical and Surgical Journal, American Medical Times, Medical and Surgical Reporter, Buffalo Medical and Surgical Journal, Transactions of Ohio State Medical Society, Circular and Catalogue of the Long Island College Hospital, The Dental Register, Catalogue of the Berkshire Medical Institute for the year 1863.

